# The multifaceted role of mitochondria in autism spectrum disorder

**DOI:** 10.1038/s41380-024-02725-z

**Published:** 2024-09-02

**Authors:** Igor Khaliulin, Wajeha Hamoudi, Haitham Amal

**Affiliations:** Institute for Drug Research, School of Pharmacy, Faculty of Medicine, The Hebrew University of Jerusalem, Jerusalem, Israel

**Keywords:** Biochemistry, Diseases

## Abstract

Normal brain functioning relies on high aerobic energy production provided by mitochondria. Failure to supply a sufficient amount of energy, seen in different brain disorders, including autism spectrum disorder (ASD), may have a significant negative impact on brain development and support of different brain functions. Mitochondrial dysfunction, manifested in the abnormal activities of the electron transport chain and impaired energy metabolism, greatly contributes to ASD. The aberrant functioning of this organelle is of such high importance that ASD has been proposed as a mitochondrial disease. It should be noted that aerobic energy production is not the only function of the mitochondria. In particular, these organelles are involved in the regulation of Ca^2+^ homeostasis, different mechanisms of programmed cell death, autophagy, and reactive oxygen and nitrogen species (ROS and RNS) production. Several syndromes originated from mitochondria-related mutations display ASD phenotype. Abnormalities in Ca^2+^ handling and ATP production in the brain mitochondria affect synaptic transmission, plasticity, and synaptic development, contributing to ASD. ROS and Ca^2+^ regulate the activity of the mitochondrial permeability transition pore (mPTP). The prolonged opening of this pore affects the redox state of the mitochondria, impairs oxidative phosphorylation, and activates apoptosis, ultimately leading to cell death. A dysregulation between the enhanced mitochondria-related processes of apoptosis and the inhibited autophagy leads to the accumulation of toxic products in the brains of individuals with ASD. Although many mitochondria-related mechanisms still have to be investigated, and whether they are the cause or consequence of this disorder is still unknown, the accumulating data show that the breakdown of any of the mitochondrial functions may contribute to abnormal brain development leading to ASD. In this review, we discuss the multifaceted role of mitochondria in ASD from the various aspects of neuroscience.

## Introduction

Autism spectrum disorder (ASD) is a neurodevelopmental condition representing one of the most disabling chronic disorders in childhood [1]. It displays abnormalities in social interactions, restricted interests, deficits in communication, and repetitive behavior [2]. ASD children are often subjected to bullying [3] and react with hostility, isolation, or even self-harm [4]. These features are aggravated by an inflexible adherence to routines and inadequate reaction to sensory stimulation. Gradually, this disorder descends into a permanent lifelong disability [5]. Recent studies determined that the global prevalence rate of ASD is 1 in 36 children [6]. The last decade has been characterized by a dramatic increase in the number of children diagnosed with ASD. It has been found that the prevalence rate of children with ASD in the US grew by 52% between 2017 and 2020 [7]. ASD is likely to affect the entire family of the person diagnosed with this disorder due to a significant level of stress associated with the permanency of this disorder, the accompanying co-morbidities, and insufficient health support for autistic patients [8, 9]. Finding the markers and therapeutic targets for ASD treatment will significantly impact the global economy.

ASD has a diverse etiology. However, it is believed that common mechanisms underlying the behavioral deficits of ASD can be found [10]. Targeting these mechanisms may result in novel therapeutic approaches aimed at developing the means of effective prevention and treatment of the core ASD symptoms [11]. One organelle containing such targets could be mitochondria. Mitochondria comprise many common molecular pathways and are the main energy source for the brain tissues. On top of its important role as the “powerhouse of the cell,” these organelles are also essential regulators of cellular metabolism, redox state, intracellular calcium signaling, and programmed cell death mechanisms [12–15]. It has been shown that mitochondrial functions are often disrupted in ASD patients [16]. This could be partly associated with the mitochondrial DNA (mtDNA) mutations in autistic children [16]. Mitochondrial dysfunctions in ASD individuals could also result from various risk factors, both endogenous and exogenous, including toxins, immune stimulation, drugs, and metabolic abnormalities [17]. Thus, ASD is tightly associated with changes in mitochondrial structure and functions, which make these organelles a likely end effector in ASD patients with different etiology.

**Fig. 1:**
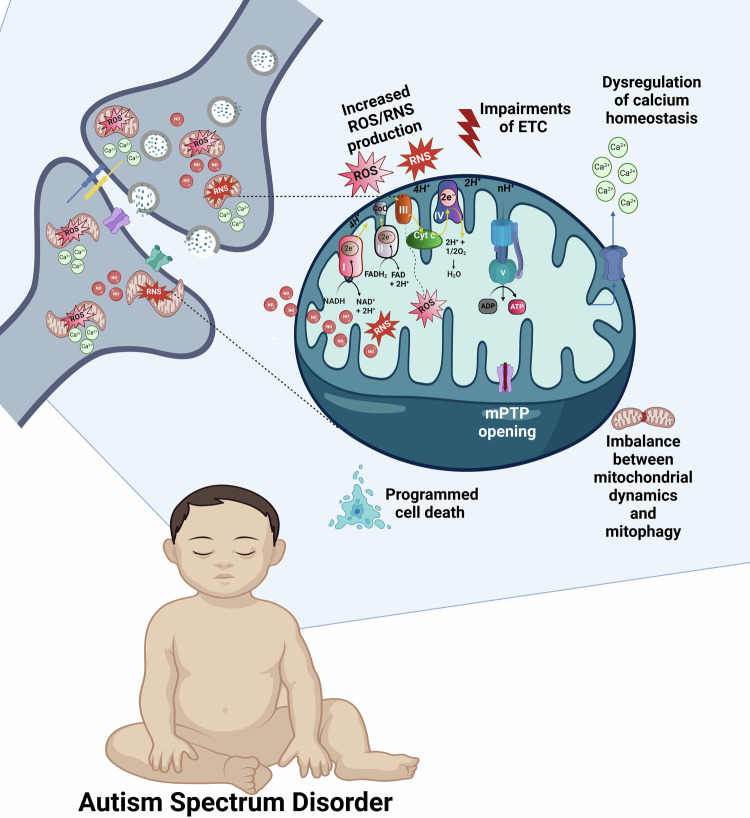
The multifaceted role of mitochondria in ASD. The brain displays high mitochondrial content, particularly in the synapses (shown in the upper left part of the figure). Increased mitochondrial levels of ROS, NO, and RNS, ETC impairments leading to the breakdown of OXPHOS and ATP production, dysregulation of the mitochondrial Ca^2+^cycling, imbalance between mitochondrial dynamics and mitophagy, prolonged opening of the mPTP, and activation of various mitochondria-related programmed cell death pathways, all contribute to the synaptic dysfunction and ASD. OXPHOS complexes are shown on the upper semisphere of the mitochondrion: I, NADH dehydrogenase; II, succinate dehydrogenase; III, ubiquinone cytochrome c oxidoreductase; IV, cytochrome c, cytochrome oxidase; and V, ATP synthase. Complexes I-IV belong to ETC.

In this review, we discuss various mitochondria-associated pathological processes related to ASD and the interplay between them. The multifaceted role of mitochondria in ASD is schematically presented in Fig. 1.

### Functions of the brain mitochondria in physiological conditions

The human brain, which weight accounts for ~2% of the body weight, consumes 20% of the whole body’s oxygen. Most of the mitochondrial oxygen is utilized for ATP production by the mitochondrial electron transport chain (ETC) through oxidative phosphorylation (OXPHOS) [18]. OXPHOS consists of two parts: the ETC and chemiosmosis. The ETC includes four complexes. Complex I is composed of NADH dehydrogenase, flavin mononucleotide, and eight iron-sulfur clusters; Complex II is succinate dehydrogenase; Complex III includes cytochrome b, Rieske subunits, and cytochrome c proteins; and Complex IV contains cytochrome *c* oxidase. Chemiosmosis is carried out by Complex V of OXPHOS, the multi-unit enzyme ATP synthase that produces ATP by utilizing the energy of the proton gradient established by ETC [19]. Importantly, 93% of the ATP necessary for normal brain functioning is supplied by mitochondria [20]. This energy supports synaptic transmission, described below, which is a very energy-demanding process. ATP provides energy for ion pumps, supporting ion gradients, to ensure vesicle recycling and mitochondrial motility.

Cytosolic Ca^2+^ entering the mitochondrial matrix through the mitochondrial Ca^2+^ uniporter (MCU) stimulates OXPHOS by activating pyruvate dehydrogenase phosphatase, which in turn, increases the activity of pyruvate dehydrogenase complex [21]. Other mitochondrial citrate cycle enzymes, such as alpha-ketoglutarate dehydrogenase and isocitrate dehydrogenase can also be activated by Ca^2+^ [21, 22]. Mitochondria can release Ca^2+^ into the cytosol via the mitochondrial permeability transition pore (mPTP) or the Na^+^/Ca^2+^ exchanger [23, 24]. This results in the accumulation of cytosolic Ca^2+^ followed by activation of synaptic vesicle exocytosis, and release of neurotransmitters.

Mitochondria are also the main source of reactive oxygen species (ROS) and reactive nitrogen species (RNS). ROS and RNS production in physiological conditions is maintained at low levels [25, 26] and used to regulate various physiological functions, including cell signaling, homeostasis, vascular tone, and immune reactions [27]. They include various free radicals and reactive molecules formed from molecular oxygen and nitrogen, including superoxide anion radical (O^•–^), hydroxyl radical (OH^·^), hydrogen peroxide (H_2_O_2_), singlet oxygen (^1^O_2_), nitric oxide (NO), and peroxynitrite (ONOO^−^) [28]. ROS and RNS may trigger programmed cell death, e.g., apoptosis [29], necessary for tissue remodeling during the development and normal tissue turnover [30], proliferation, differentiation, maturation of neural stem cells, dendritic processes, and synaptic plasticity [31–33].

Since the developing nervous system and its critical processes rely heavily on the ATP produced by OXPHOS in the mitochondria, the immature brain is critically vulnerable to faults in energy supply [34, 35]. Moreover, under pathological conditions, from a life-supporting organelle, the mitochondrion becomes an organelle that actively supports death [36]. This process is accompanied by the ETC breakdown with the failure to produce the necessary amount of ATP, uncompensated production of ROS and RNS, and breakdown of various molecules, which leads to the loss of mitochondrial and cell integrity, and activation of programmed cell death and necrosis. All these outcomes of mitochondrial dysfunction may disrupt the neurodevelopmental processes, and it is not surprising that mitochondrial impairments are implicated in neurodevelopmental disorders, such as ASD [34]. Moreover, it has been found that mitochondrial functions and integrity can be affected by many of the risk factors of ASD, including toxins, immune activation, medicines, and metabolic disturbances [17]. Uncovering these mitochondria-related mechanisms may shed light on the common pathways of ASD [11].

### Role of mitochondria in synaptic transmission

Most of the energy, produced by the brain mitochondria [18], is used for synaptic transmission [20]. This determines the crucial role of mitochondria in brain functioning. Interestingly, the number of mitochondria in synapses is very high. It exceeds the predicted energy demand [20]. This means that the synapse mitochondria have additional functions at the nerve terminals, which may include Ca^2+^ buffering directly affecting the firing probability of neurons [37, 38].

Previous studies suggest two main pools of mitochondria: a motile and an immobile [39, 40]. It is generally accepted that in developing neurons each pool covers around 50% of the mitochondria [41]. However, with the maturation of neurons, mitochondrial trafficking significantly declines [42, 43]. Mitochondrial transport and distribution correlate with synaptic activity [44]; their accumulation at presynaptic terminals and postsynaptic dendritic spines is increased when the synaptic activity is elevated [31]. Mitochondrial transport is carried out by a special mechanism involving the cytoskeleton. It is mediated by kinesin-1, which binds to mitochondria via anchoring proteins including the microtubule-associated motors dynein-dynactin, the Ca^2+^-dependent mitochondrial Rho GTPase 1 (Miro1), and the trafficking kinesin proteins 1 and 2 [45]. It has been suggested that the first step in blocking mitochondria trafficking along the axon is carried out by the Ca^2+^-dependent release of mitochondria from microtubules involving the combined actions of the key adaptors of kinesins and dyneins, Miro/Milton, and syntaphilin (SNPH) [46, 47]. Lin et al. have found in experiments on primary cortical neurons that SNPH also promotes the removal of damaged mitochondria in axons independently of mitophagy by creating vesicles with late-endosomes [48]. Other, yet unknown, mechanisms might also contribute to the “anchoring” of mitochondria at axon terminals or other locations [41].

Mitochondrial morphology depends on their localization in the two neuronal compartments, the axon, and dendrites [41]. For example, it has been found that in the cortical pyramidal neurons, dendritic mitochondria are presented in long, tubular-shaped form, occupying 70–80% of the dendritic branches. Meanwhile, axonal mitochondria have a remarkably standard small size, taking less than 10% of axonal volume [49, 50]. This morphological variability likely affects mitochondrial functions, such as ATP production and Ca^2+^ buffering. However, the effect of these differences on neuronal development and function remains unknown [41].

The involvement of mitochondria in Ca^2+^ cycling regulation and ATP production makes these organelles crucial for synaptic function during neurodevelopment and in the adult brain. Exocytosis of synaptic vesicles (SV) containing neurotransmitters is initiated by Ca^2+^ influx through voltage-gated Ca^2+^ channels during excitation of the presynaptic bouton. This neurotransmitter’s release is coupled to the endocytosis of SV to preserve the SV pool for the normal functioning of the synapse. Presynaptic cytoplasmic Ca^2+^ can be depleted by several complementary mechanisms, including sarcoendoplasmic reticulum Ca^2+^-ATPase (SERCA) to the endoplasmic reticulum (ER), plasma membrane Ca^2+^-ATPase to the extracellular space, and MCU to the mitochondrial matrix [51–53]. Studies with genetically encoded sensors for Ca^2+^ cycling and SV exocytosis revealed the core roles of mitochondria in presynaptic Ca^2+^ clearance [54, 55]. It has been shown that mitochondria-free axon terminals of hippocampal or cortical neurons accumulate more Ca^2+^ in response to repetitive stimulation, which promotes increasingly more SV release [55, 56]. Here too, inhibition of MCU-dependent presynaptic Ca^2+^ uptake caused increased presynaptic cytoplasmic Ca^2+^ levels and negatively affected short-term synaptic plasticity [55]. Remarkably, it has been demonstrated that mitochondria can be recruited to presynaptic boutons in response to elevated neuronal activity and contribute to rescaling synaptic excitability to match the neuronal stimulation [56].

It has been generally appreciated that ATP production by the mitochondria is critically important presynaptically [45]. It is needed for neurotransmitter reuptake, endocytosis of SV, and presynaptic vesicles, let alone maintain the concentration gradients of ions across the membrane. This opinion is supported by a study showing activity-driven ATP production at axon terminals [57]. However, Lee et al. have found that even at non-physiologically high action potential stimulations, inhibition of glycolysis or the mitochondrial ATP synthesis leads to only mild changes in presynaptic ATP levels [41]. Another study compared changes in ATP levels in axon terminals with or without mitochondria. They observed no difference in this parameter between the presynaptic boutons even under high-intensity stimulation of neurotransmitter release (600AP) [58]. These data imply that in the mammalian axons of the adult brains, presynaptic mitochondria are probably not the main source of ATP. The authors suggest that glycolysis or other ways of ATP production may supply sufficient ATP necessary for synaptic activity [41].

Most studies on the role of mitochondria in synaptic transmission have focused on the organelle’s presynaptic pool. Meanwhile, the role of postsynaptic mitochondria is less investigated. Mitochondria are mainly found in the dendritic shafts but can also reach the spines [59]. The importance of the dendritic pool of mitochondria has been confirmed by the fact that in hippocampal neurons, the depletion of mitochondria in dendrites diminishes the number of synapses and spines [31]. Dendritic postsynaptic Ca^2+^ cycling is important for synaptic integration and regulation of gene expression [41]. The main sources of dendritic Ca^2+^ are ER and extracellular space. However, studies using 3D-serial electron microscopy demonstrated that dendritic ER has many contact sites with mitochondria (MAMs, mitochondria-associated membranes) [60]. MAMs are essential for the regulation of the neuronal Ca^2+^ concentration via the SERCA, the ER channels inositol 1,4,5-trisphosphate receptors, the glucose-regulated protein 75, the MCU, and the voltage-dependent anion channel (VDAC) [61]. Furthermore, it appears that a significant portion of Ca^2+^ released from the synaptic ER is directly transported to mitochondria at the MAMs [62]. In dendrites of cortical pyramidal neurons lacking a novel MAM protein PDZ domain-containing protein 8, a significant fraction of Ca^2+^ released from the ER during synaptic stimulation accumulates in the cytosol and elevates the local dendritic Ca^2+^ levels. These data imply that the distribution and extent of MAMs in the dendrites may regulate Ca^2+^ dynamics and thus, determine the properties of synaptic integration and plasticity at the dendrites [62].

Synaptic developmental abnormalities appear to be essential contributors to ASD. To emphasize the importance of these aberrations in ASD, this disorder is now referred to as “developmental synaptopathy” [63]. It should be noted that the abnormalities of synaptic transmission in ASD are tightly associated with mitochondrial aberrations. The synaptic and mitochondrial aberrations in different ASD-related syndromes are discussed below in the section “Mitochondrial and synaptic abnormalities in ASD-related syndromes”.

### Mitochondrial dysfunction in ASD

Metabolic studies have linked mitochondria to the pathophysiology of ASD [64]. Back in 1985, Blass and Coleman reported on the increased levels of lactate in the plasma of four autistic patients and suggested that this was a result of aberrations in OXPHOS [65]. Thirteen years later, Lombard reviewed the data on the metabolic changes in ASD patients. He hypothesized that lactic acidosis, increased concentration of Krebs cycle metabolites in urine, reduced levels of carnitine in plasma, decreased utilization of glucose in the brain, and lowered ATP levels in these patients are associated with mitochondrial dysfunction [66]. Based on these data, Lombard proposed that ASD is a mitochondrial disease [66]. The data on metabolic abnormalities in ASD patients continue to accumulate (these data are summarized in Table 1). Thus, the levels of mitochondria-related metabolites, such as pyruvate, carnitine, and ubiquinone in the blood of children diagnosed with ASD appeared to be significantly different from those of their typically developing peers [35]. Correia et al. have found high levels of lactic acid in the plasma of 17% of the studied cohort of ASD children, and 28% of them displayed increased levels of lactate/pyruvate ratio [67]. Muscle biopsies taken from 30 autistic children revealed a mitochondrial defect in 7 of these children [68]. Further studies supported these findings. For example, in 8.3% of the 60 ASD patients, biochemical markers of failed aerobic respiration were found [69]. These markers included increased plasma alanine and lactate levels and the presence of organic acids, such as 3-methyl-glutaconic and dicarboxylic acids, and Krebs cycle intermediates, in the urine of these patients [69]. In another work, 20% of ASD children displayed increased plasma lactate levels and a lactate/pyruvate ratio [70]. A decrease in free and total serum carnitine concentration, reduced pyruvate, and increased alanine and ammonia levels were observed in another cohort of patients diagnosed with ASD [71]. A review of the medical examination data from 25 autistic children found that 53% of these patients had increased pyruvate levels, 76% had elevated blood lactate, in 20% of them the lactate/pyruvate ratio in fibroblasts was increased, and 42% were presented with atypical results of urine organic acid analysis [72].

**Table 1:** Impairments of the mitochondrial respiratory function in ASD patients.

Source (organs/tissues)	Abnormalities in ASD related to mitochondrial respiration	References
Blood	• Increased levels of lactate • Increased lactate/pyruvate ratio • Increased levels of pyruvate • Decreased levels of pyruvate • Decreased activity of pyruvate dehydrogenase • Reduced levels of carnitine • Increased levels of carnitine • Elevated levels of alanine • Increased ammonia levels	[65–67, 69, 70, 72] [67, 70, 72] [72] [71] [81] [35, 66, 71] [72] [69, 70] [70]
Urine	• Increased concentration of Krebs cycle metabolites • Presence of 3-methyl-glutaconic acid • Presence of dicarboxylic acids	[66, 69, 72] [69, 72] [69, 72]
Brain	• Decreased utilization of glucose • Reduced ATP levels • Increased levels of NO	[66] [66] [64, 149]
Skeletal muscle	• Enhanced activity of complex I • Defects of complex I (in 50% of ASD patients) • Combined defects of complexes I and III (in 18% of ASD patients) • Defects of complex V (in 14% of ASD patients) • Aberrations in OXPHOS (in 71% of ASD patients) • Defects in complexes I, III, IV, and V	[73] [74] [74] [74] [74] [77]
Lymphocytes	• Defects in complexes I, III, IV, and V	[75]
Brain	• Decreased activity of complexes I and V (in 30% of ASD patients) • Reduced activity of complex III (in 29% of ASD patients) • Reduced activity of multiple complexes (in 29% of ASD patients)	[81] [81] [81]

Evidence of OXPHOS impairments in ASD patients, including the disruptions of ETC activity, has also been gathered (Table 1). The results of these studies are rather ambiguous. Thus, Graf et al. have reported abnormally enhanced complex I activity in mitochondria obtained from a skeletal muscle biopsy of an ASD patient [73]. Meanwhile, Shoffner et al. have found that among the skeletal muscle biopsies of 28 ASD children with mitochondrial diseases, 50% had defects of complex I, 18% had combined defects of complex I and III, another 18% were identified with the combined defects of complexes I, III, and IV, and 14% were with defects of complex V [74]. Seventy-one percent of these children had abnormal OXPHOS. Defects in complexes I, III, IV, and V were also reported in a few other studies on the mitochondria isolated from autistic children [75–77]. The study on the brains of the *Mecp2-308* mouse model of ASD has shown a reduced ATP production accompanied by a significant reduction in complexes I, II, and V activities in the cerebellum and striatum of the mutant mice [78]. These findings indicate that the most affected component of OXPHOS in ASD patients is complex I, but abnormalities in the activity of other complexes, such as complexes III, IV, and V, can also be found.

Clinical studies of ASD and experiments on animal and cellular models of this disorder have revealed the biochemical endophenotype of insufficient mitochondrial energy production. This phenotype was manifested in the accumulation of lactic acid, pyruvate, and carnitine, increased alanine aminotransferase and aspartate aminotransferase levels in plasma [72], suppressed ETC activity, and reduced mitochondrial membrane potential (Ψ_m_) [79]. Age-related metabolic changes were investigated in postmortem brain samples of ASD patients by Chauhan et al. [80]. They found that the major metabolic abnormalities and increased levels of lipid hydroperoxides (the markers of oxidative stress) were observed in children of 4–10 years old but not in adults. These data suggest that autistic children of this age are particularly vulnerable to ASD-related factors such as energy deficits and oxidative stress. The following postmortem examinations of ASD patients were carried out by this group [81]. This work found a more than 30% decrease in the activities of pyruvate dehydrogenase and complexes I and V in the frontal cortex of autistic patients. Abnormal activity of complex III was also identified in 29% of autistic brains, and 29% of them had defects in multiple complexes [81]. Thus, some data disparity on the ASD-related defects of OXPHOS complexes can be noticed. They could stem from different methodological approaches and variability of clinical manifestations in ASD patients.

Rossignol and Frye have performed a systematic review and meta-analysis to investigate the prevalence of mitochondria-associated genetic abnormalities in autistic children [35]. This study revealed that 21% of the investigated cohort of ASD patients carried mtDNA or nuclear DNA (nDNA) mutations related to mitochondrial dysfunction. Other population-based studies have found that ~7% of ASD patients had an OXPHOS dysfunction [70, 72]. In 23% of these patients, mtDNA abnormalities were observed [35]. It is not known whether the mtDNA mutations in autistic patients are a cause or effect of ASD. However, a functional role of mitochondrial disease in ASD phenotype has been proposed by some researchers [74]. One of the best-known mitochondrial diseases is mitochondrial encephalomyopathy, lactic acidosis, and stroke-like episodes (MELAS) [82]. It has been found that MELAS results from the A3243G mtDNA mutation which appears to be linked to autism [83].

OXPHOS and mitochondrial integrity can also be affected by the defects in the expression of nDNA encoding the mitochondrial proteins. Filipek et al. have identified two cases of ASD children with an inverted duplication of chromosome 15q11-q13. The authors suggested that the gene products of this chromosome are involved in complex III regulation [84]. Genes in other nDNA regions responsible for mitochondrial proteins may also be implicated in the ASD phenotype. Thus, in a cohort of 235 ASD patients, the 7q32 region, a candidate ASD region, was investigated [85]. In this region, two single-nucleotide polymorphisms within the *NADH-ubiquinone oxidoreductase 1 alpha subcomplex 5 (NDUFA5)* gene were strongly associated with autism. NDUFA5 is a part of complex I of the ETC and its mutations may contribute to ASD [86]. Wang et al. have analyzed the whole exome from 903 autistic proband-mother-sibling trios [87]. They found that the likelihood of heteroplasmic mutations in non-polymorphic sites, the sites that may produce OXPHOS abnormalities, is 53% higher in ASD children than in unaffected siblings. Contrary t o this study, however, the mitochondrial genome sequencing of ∼400 proband-father pairs found no evidence of a link between mtDNA mutations and ASD [88]. Collectively, genetic mutations related to mitochondrial dysfunctions have been identified in autistic children. A significant data disparity can be noticed in studies on ASD-related genetic mutations, which can be associated with the diversity of the autistic spectra. Whether these mutations have a causative or associative role in the ASD phenotype remains to be determined.

Taken together, unmistakable evidence has been gathered that points to a strong link between autism and mitochondrial respiratory dysfunction (summarized in Table 1).

### Role of mitochondrial Ca^2+^ in ASD

Calcium signaling is a critical regulator of various mitochondria-related cellular processes and functions in physiological settings. Normally, the distribution of Ca^2+^ in the cell and the intercellular space is regulated by the mechanisms of Ca^2+^ homeostasis, and mitochondria play a key role in these processes [89]. When cytosolic Ca^2+^ levels are increased, mitochondria become a high-capacity storage for these ions contributing to the normalization of Ca^2+^ concentration [89]. Accumulation of Ca^2+^ in mitochondria stimulates the Krebs cycle and ATP production by OXPHOS [90]. Interestingly, it has been found that extramitochondrial Ca^2+^ also regulates mitochondrial metabolism. This is achieved by transporting glutamate to the mitochondrial matrix through a mitochondrial aspartate/glutamate carrier aralar [91, 92]. As discussed above (in the section “Role of mitochondria in synaptic transmission“), Ca^2+^ signaling is involved in the accumulation of mitochondria at the postsynaptic regions, where these organelles participate in neuronal Ca^2+^ buffering and support of neurotransmission [93]. Ca^2+^ is implicated in the regulation of neurotransmitter release from presynaptic nervous terminals. Meanwhile, the neurotransmitters gamma-aminobutyric acid (GABA) and glutamate participate in Ca^2+^ signaling at postsynaptic neurons [93, 94]. For example, ionotropic glutamate receptors represent ligand-gated calcium channels while GABA receptors trigger calcium influx via voltage-gated calcium channels [94]. Hence, the subcellular distribution of Ca^2+^ determines the fine regulation of Ca^2+^ signaling [95].

In neurodevelopmental conditions, including ASD, disruption of Ca^2+^ homeostasis may cause detrimental effects on various cellular processes [15]. Ca^2+^ is not metabolized and an overload of mitochondrial Ca^2+^ may break down the electrochemical proton gradient resulting in a deficit of ATP followed by necrosis [89]. Mitochondrial Ca^2+^ overload also triggers apoptotic cell death by increasing ROS production and opening the mPTP [96]. Impaired Ca^2+^ homeostasis can affect migration, proliferation, Purkinje cell development, dendritic arborization, synapse formation, and maintenance [94]. In addition, aberrant Ca^2+^ signaling causing mitochondrial dysfunction can adversely affect neurotransmitter signaling and lead to excitation/inhibition imbalance [94, 97, 98]. All these adverse effects may contribute to ASD.

One of the triggers of Ca^2+^ release from the brain mitochondria can be perisynaptic ATP bound to astrocyte receptors. These ATP molecules cause depolarization of the inner mitochondrial membrane (IMM) and generation of ROS [93]. Also, the release of Ca^2+^ from the mitochondria can be mediated by extracellular ATP bound to microglial purinergic receptors. The abnormal release of Ca^2+^ from the mitochondria to the cytosol leads to the activation of microglia, neuroinflammation, and finally, cell death [29, 93]. Altogether, the dysbalanced regulation of mitochondrial and cytosolic Ca^2+^ cycling contributes to ASD pathogenesis by causing mitochondrial dysfunction, cell signaling breakdown, cytotoxicity, and oxidative stress. Here, we discuss the role of oxidative/nitrative/nitrosative stress in ASD pathogenesis.

### Mitochondrial ROS in ASD

ROS, at low levels, regulate various physiological functions, including autophagy, immune system, cell differentiation, cell survival, programmed cell death, and adaptation to hypoxia [27, 99]. Normally, the excess of ROS in the mitochondria is neutralized by the endogenous antioxidant system which comprises several enzymes and non-enzymatic antioxidants [100] including reduced glutathione (GSH), vitamins C, and E, the Cu/Zn-superoxide dismutase (SOD) in the cytoplasm, and Mn-SOD in the mitochondrial matrix, catalase in the peroxisomes, glutathione peroxidase (GSH-Px), etc. Different isoforms of GSH-Px are present in mitochondria. The mitochondrial GSH-Px1 converts H_2_O_2_ to H_2_O via oxidation of GSH to oxidized glutathione (GSSG) [101]. GSH-Px4 can also be found in the mitochondria. It neutralizes lipid hydroperoxides, products of oxidative damage to the membrane phospholipids. Peroxiredoxin (Prx) 3, a cysteine-dependent peroxidase enzyme, is also present in the mitochondria, whereas Prx5 can also be found in the cytosol, in peroxisomes, and in the nucleus [28]. The function of Prxs is to oxidize H_2_O_2_ to cysteine–SOH. Cysteine-SOH interacts with another cysteine, producing H_2_O and a disulfide bond. The latter can be reduced by thioredoxin (Trx) 2, which is further reduced by thioredoxin reductase [28].

GSH and other mitochondrial non-enzymatic antioxidants and antioxidant enzymes determine the antioxidant capacity of the cell [102]. Weakening of the endogenous antioxidant system leads to uncontrolled ROS production that outweighs the antioxidant capacity of the brain, causing developmental neurotoxicity. The brain’s restricted antioxidant capacity, high energy demand, high levels of transition metals, such as iron and copper, and a high concentration of polyunsaturated fatty acids which can be subjected to lipid peroxidation [103–105] make the nervous system particularly vulnerable to oxidative stress [106]. Therefore, neurons are the first cells that appear to be affected by oxidative stress [28].

Oxidative stress is involved in a variety of neurodegenerative diseases, such as Alzheimer’s disease (AD), Huntington’s, and Parkinson’s diseases [107–109], and neurodevelopmental disorders, including ASD [28, 110, 111]. Signs of oxidative damage to proteins, lipids, and DNA have been found in blood [35, 112], urine [113], and post-mortem brain samples [79, 80] collected from autistic individuals. For example, markers of enhanced oxidative stress and diminished methylation ability, such as reduced S-adenosylmethionine/S-adenosylhomocysteine and GSH/GSSG ratios, have been detected in the plasma of autistic children [114–116]. Increased lipid peroxidation marker concentrations, 8-isoprostane-F_2*α*_, have also been found in ASD patients [113]. These results were consistent with the findings of elevated urinary levels of the marker of lipid peroxidation, isoprostane F_2*α*_-VI, the marker of platelet activation, 2,3-dinor-thromboxane B_2_, and the marker of endothelial activation, 6-keto-prostaglandin F_1*α*_ in 26 children diagnosed with ASD [117]. Changes in the levels of plasma biomarkers corroborate with the results of postmortem studies on ASD patients. For example, accumulation of lipid hydroperoxides [80], and increased oxidative DNA damage accompanied by reduced levels of SOD [79] were observed in the brain of autistic individuals. Here too, a decreased activity of glutathione-S-transferase, GSH-Px, and glutamate cysteine ligase was found in the postmortem examination of the cerebellum of autistic children [118]. Manifestations of oxidative stress have also been reported in the hippocampus and temporal cortex of ASD patients [119, 120].

The main source of ROS in the cell is the mitochondrial OXPHOS [121]. Post-mortem studies of the autistic brain samples showed abnormal changes in the steady-state levels of complexes I-IV in the cingulate gyrus, cerebellum, thalamus, and temporal and frontal cortex [79, 80, 86, 122]. The levels of complexes III and V were found to be decreased in autistic patients. Others found that complexes II, III, and V in the temporal cortex and complex I in the frontal cortex were downregulated in ASD subjects [80]. In another work, Brodmann area 21 of the lateral temporal lobe was investigated in ASD patients [79]. Brodmann area is important for ASD symptoms because it is implicated in the processing of language, auditory, and social perception [79]. This study also identified decreased concentrations of complexes I, III, IV, and V and weakened activities of complexes I and IV [79]. Another postmortem study has supported these findings by reporting a decreased expression of some subunits of complex I, III, IV, and V in the areas of the cingulate gyrus, motor cortex, and thalamus of the ASD subjects [122]. Downregulation of ATP5A1, the ATP Synthase F1 Subunit Alpha [123], and ATP5G3, the ATP synthase F0 complex subunit C3 [124], was observed in the postmortem study of ASD patients in all examined regions [122], which could inhibit the activity of complex V [79] and promote the ETC breakdown due to opening of the mPTP [125, 126]. In contrast, it has been reported that in the neurons of mice with Fragile X syndrome (FXS), ATP5G1, the *c* subunit of F_0_-ATP synthase, is abnormally upregulated. This also results in the opening of the mPTP leading to uncontrolled ROS production and disrupted synaptic maturation causing autistic behaviors [127]. Thus, the aberrant activity of the F_1_F_0_-ATP synthase might be implicated in ASD via the disrupted functioning of ETC. Overall, complex I appears to be most affected in autistic patients whereas complex II is least affected [128]. Dysfunctional ETC, in turn, further enhances ROS production and increases the disruption of the mitochondrial respiratory function and mitochondrial integrity [129]. Thus, unneutralized ROS produced in the brain mitochondria of autistic individuals form a positive feedback, a vicious cycle, leading to incrementally growing damage to the mitochondria, which may ultimately result in cell death.

It is worth noting that children possess a weaker antioxidant defense than adults [28, 130]. This makes oxidative stress a big risk factor for ASD. It has been revealed that SOD activity in erythrocytes of ASD children is significantly increased compared to their typically developing peers [131]. The authors explained this fact by a compensatory mechanism to counter the detrimental effects of oxidative stress within the brain. Indeed, it has been found that children diagnosed with ASD have reduced levels of mitochondrial GSH and impaired mitochondrial respiratory function due to oxidative stress [35, 119, 132]. Various oxidative stress markers, including lipid peroxide [80], malondialdehyde [133], a marker of oxidative DNA damage 8-hydroxy-2’-deoxyguanosine [119], and protein carbonyls [134, 135] are increased in ASD children. It has been revealed that oxidative stress is involved in neuro-inflammation [136], cerebral injury [137, 138], and neuro-dysfunction [136–138], leading to neurodevelopmental disorders. Thus, the accumulating data indicate that ROS contribute to ASD phenotype, although the mechanisms of the oxidative injury and the weakening of the antioxidant system remain obscure.

### Role of reactive nitrogen species in ASD

Along with ROS, another important family of redox-active molecules related to oxidative stress is reactive nitrogen species (RNS). Nitric oxide (NO) is a free radical gas molecule produced endogenously from L-arginine, oxygen, and NADPH by an enzyme nitric oxide synthase (NOS) [139]. Three isoforms of NOS, neuronal (nNOS), inducible (iNOS), and endothelial (eNOS) have been identified. At low concentrations in physiological conditions, NO production and inactivation are balanced [140]. NO is involved in normal cell signaling, contributing to the regulation of various physiological functions [141, 142] including activation of soluble guanylyl cyclase (sGC) which generates cyclic GMP (cGMP) [143]. NO may exert therapeutic effects on the injured brain [144]. This molecule can also stimulate mitochondrial biogenesis in different organs, including the brain [145, 146]. However, at high concentrations, when RNS levels exceed the capability of its detoxification in the biological system, serious damage to cells may occur [147, 148] due to inhibition of mitochondrial respiratory function by competing with O_2_ for interaction with cytochrome oxidase [149] and via nitrative/nitrosative stress [150, 151].

Nitrative stress is directly related to oxidative stress. NO can form peroxynitrite (ONOO^−^) by reacting with O^•–^ in the mitochondria [152]. ONOO^−^ is a highly reactive molecule. It can destroy lipids, DNA, and protein, and trigger apoptosis by inducing cytochrome *c* release from the mitochondria [153]. The OXPHOS functioning may also be affected by ONOO^−^ because it can compete with oxygen for binding cites [154]. As a result, more ROS and RNS are produced by the mitochondria. Autoxidation of the mitochondrial NO can generate nitrogen dioxide (^•^NO_2_) [140]. ^•^NO_2_ and ONOO^−^ can nitrate various molecules. In proteins, tyrosine residues are often subjected to nitration with the formation of 3-nitrotyrosine (NT) [155]. NT disrupts the hydrogen bonds of proteins, impairing protein function [156, 157]. Nitrative stress is common to ASD as seen by elevated levels of NT in autistic patients [158].

Another important form of NO-related posttranslational modification (PTM) is protein S-nitrosylation (SNO), a product of the interaction of NO with the sulfhydryl groups of cysteine which leads to the formation of S-nitrosothiols [147, 159]. Normally, SNO is maintained in the brain at a low level. It participates in the modulation of the activity and localization of numerous enzymes and receptors [147, 160, 161], takes part in the regulation of many physiological processes in the brain [147, 162], including synaptic plasticity [163, 164], axonal elongation, and neuronal survival [147, 165]. This PTM also occurs in mitochondria, regulating the OXPHOS and other mitochondrial functions [166]. However, various neurodevelopmental disorder conditions, including ASD, may cause steadily high levels of NO in the brain, promoting abnormal SNO of mitochondrial proteins. This aberrant SNO can lead to conformational changes and misfolding of the proteins that affect their functions [167]. As a result, the aberrant protein SNO may significantly affect neuronal functions, thereby contributing to behavioral deficits in ASD.

In 1998, Lombard hypothesized that mitochondrial dysfunction in ASD patients could be associated with excessive NO production causing neurotoxicity [66]. Based on the work of Hibbs, et al. [168], he proposed that NO may bind to the enzymes of mitochondrial ETC, such as NADH succinate oxidoreductase, NADH ubiquinone oxidoreductase, and cis aconitase and induce uncoupling of OXPHOS followed by the inhibition of glycolysis [66] and depletion of ATP in the cells [169]. The inhibition of mitochondrial respiration leads to depolarization of the IMM, followed by the opening of the mPTP and activation of apoptosis [170].

Lombard’s hypothesis of the role of NO in ASD was confirmed later in the animal models of ASD and autistic patients. Thus, we have developed the SNOTRAP technology to study SNO-proteome. Using this technique, we found a significant increase in NO levels and a reprogramming of the SNO-proteome in the brain of *Shank3* InsG3680^(+/+)^ mouse model of ASD [171]. Our later studies on the *Shank3*^*-/-*^ and *Cntnap2*^*-/-*^ knockout mice, the human SH-SY5Y cell line, and the human induced pluripotent stem cells‐derived cortical neurons isolated from patients carrying a *SHANK3* mutation confirmed the increased levels of NO and protein SNO [172–176]. Importantly, this work also revealed the synaptic and behavioral abnormalities in these models of ASD [172, 173, 175] that were reversed by the selective nNOS inhibitor 7-nitroindasole [172, 173]. Our results were in line with the postmortem data showing increased levels of NO in plasma [177] and NT, the marker of nitrosative stress [178], in the brain of autistic patients [179]. These data represent evidence of the involvement of excessive levels of NO in the pathogenesis of ASD.

Numerous mitochondrial proteins contain essential thiol residues. These thiols form S-nitrosoglutathione (GSNO) and SNO upon direct interaction with NO and by transnitrosation reactions with other SNO proteins [180]. Our recent SNO-proteome analysis of the cortices of *Shank3* InsG3680^(+/+)^ mutant mice showed that several mitochondrial processes, including the ATP metabolic process, transmembrane electron transport, and the cellular response to oxidative stress, were SNO-enriched [64]. The aberrant SNO of the mitochondrial proteins is likely to lead to mitochondrial dysfunction and contribute to ASD pathogenesis.

Overall, the above studies point to the significantly increased levels of NO and products of its redox reactions in the mitochondria in the ASD animal models and autistic patients. Along with oxidative stress, these abnormal molecular changes lead to impaired mitochondrial respiratory function, ATP depletion, and various destructive cellular processes, such as mitochondrial fission, mitophagy, and programmed cell death, as we discuss below. These data indicate that NO-associated redox reactions occurring in the mitochondria play an important role in ASD, although the exact mechanisms implicating NO in this role need further investigation.

### Mitochondrial permeability transition pore in ASD

Under physiological conditions, the IMM is impermeable to most molecules and ions. Only a few essential ions and metabolites can move through the pores of the IMM, thereby maintaining the Ψ_m_ and pH gradient required for ATP synthesis by OXPHOS. [181]. However, this membrane contains a non-specific pore, called the mPTP, which allows any molecule of <1.5 kDa to move freely across the IMM [182]. The exact molecular structure of this pore remains obscure and its physiological functions have not been well established [183]. Based on the analysis of the literature on the mitochondrial Ca^2+^ regulation in cyclophilin D (SypD)-deficient mice and neurons, Rizzuto et al. concluded that in physiological conditions, mPTP takes part in Ca^2+^ homeostasis by mediating Ca^2+^ efflux from the mitochondria ^15^. Normally, this pore can be open for a short time. This is now called a flickering mPTP. It is characterized by the release of small portions of Ca^2+^ and ROS from the mitochondria [184], prompting a short-term depolarization of the IMM that activates the ETC and inhibits ROS production by the mitochondria [185]. In the nervous system, Ca^2+^ release into the cytosol through the mPTP activates neurotransmitter release by triggering vesicle exocytosis [186, 187].

However, when the levels of Ca^2+^ in the mitochondrial matrix are high, especially during oxidative stress, as often observed in ASD patients, mPTP remains open for a longer time. This results in two main outcomes. First, it leads to unrestricted movement of ions through the IMM, which brings about OXPHOS uncoupling and ATP production halting. Furthermore, ATPase starts acting in reverse mode, carrying out ATP hydrolysis instead of its synthesis [181]. As a result, the ETC functioning is disrupted, and ATP content becomes significantly depleted, which breaks down metabolic and ionic homeostasis and activates the degradative enzymes such as nucleases, proteases, and phospholipases [182, 188]. Furthermore, the dysfunctional ETC produces more ROS due to incomplete oxygen reduction, damaging all kinds of mitochondrial proteins [129].

Second, non-specific permeability to any small molecules provided by the opening of the mPTP triggers mitochondrial swelling [189]. The ions and small molecules move across the IMM following the concentration gradient. This causes an osmotic imbalance between the cytosol and the matrix and increases colloidal osmotic pressure. The high molecular weight proteins of the mitochondrial matrix, which cannot move through the mPTP, also contribute to the increased colloidal osmotic pressure. As a result, the water moves into the matrix making the mitochondria swell [181]. Unfolding the cristae helps the matrix to expand and the IMM remains intact. Meanwhile, the outer membrane will rupture, and proteins, such as cytochrome *c* and apoptosis-inducing factor (AIF), will be released from the intermembrane space into the cytosol, triggering apoptosis [190, 191]. Eventually, this may result in cell death from the destructive cellular programs. Let us review the main types of programmed cell death and their roles in ASD.

### Programmed cell death in ASD

#### Apoptosis removes the damaged cells and cellular components in a highly regulated, programmed fashion

**Fig. 2:**
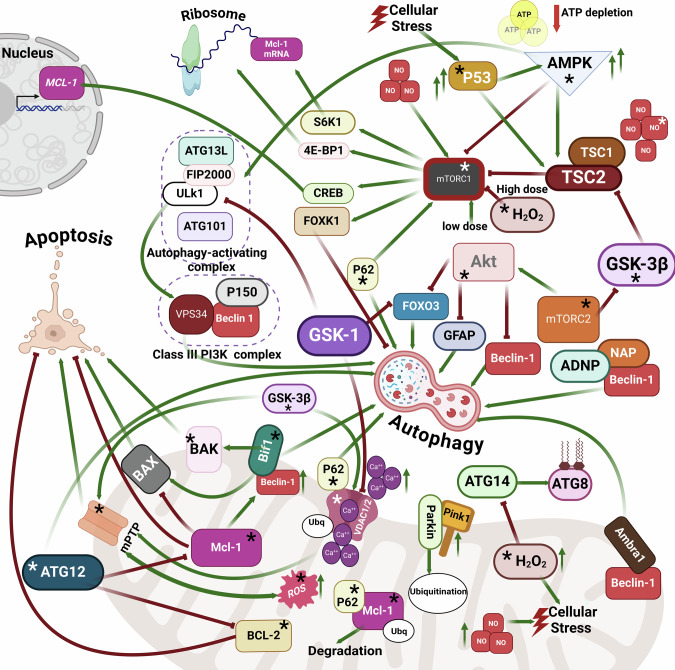
Mitochondria-related mechanisms of regulation of autophagy and apoptosis. ADNP activity-dependent neuroprotective protein, Akt protein kinase B, Ambra1 activating molecule in Beclin1-regulated autophagy, AMPK AMP-activated protein kinase, ATG autophagy-related protein, Bcl-2 B-cell lymphoma 2, Beclin1 BEC-1 in the *C. elegans* nematode, Bif1 Endophilin B1, FIP200 family interacting protein of 200 kDa, Foxk1/2 forkhead/winged helix family k1/2, FoxO3 Forkhead box O3, GFAP Glial fibrillary acidic protein, GFAP Glial fibrillary acidic protein, GSK glucose synthase kinase, Mcl-1 Myeloid cell leukemia‐1, mPTP mitochondrial permeability transition pore, NAP ADNP microtubule end binding protein motif, NO nitric oxide, OMM outer mitochondrial membrane, PI3K phosphatidylinositol-3-kinase, Pink1 PTEN-induced kinase 1, ROS reactive oxygen species, TSC1/2 tuberous sclerosis complex 1/2, Ubq ubiquitin, ULK1 unc-51-like autophagy-activating kinase 1, VDAC1 voltage-dependent anion channel 1.  Activation/upregulation/transcription.  Inhibition/downregulation. Molecules, protein complexes, and processes that regulate both autophagy and apoptosis. *, molecules and protein complexes involved in the regulation of both autophagy and apoptosis.

Apoptosis is also a necessary tool for development, including brain development. Two main pathways can trigger this process, the intrinsic, also called mitochondrial pathway, when apoptosis is initiated by the internal signals of mitochondrial origin, and the extrinsic when apoptosis is activated by external stimuli [16]. Both pathways act through a caspase activation cascade. The intrinsic pathway can be triggered either as a result of mPTP opening, or activation of the proapoptotic proteins of the B-cell lymphoma 2 (Bcl-2) family, Bax and Bak [192] after the removal of the block of antiapoptotic proteins Bcl-2 and Bcl-XL [193]. This makes the outer mitochondrial membrane (OMM) permeabilized. The mitochondrial membrane permeabilization results in the release of the proapoptotic factors from the mitochondrial intermembrane space, cytochrome *c* [194], AIF [195], and endonuclease G [196], to the cytosol. Cytochrome *c*, apoptosis protease activating factor 1 (APAF-1), and pro-caspase 9 form a protein complex called apoptosome [197]. The apoptosome activates caspase 9, which activates effector caspases, leading to the completion of apoptosis (Fig. 2). AIF [195] and endonuclease G [198] trigger DNA fragmentation followed by chromosomal condensation.

Apoptosis is an essential process for normal brain development. Meanwhile, abnormal apoptosis may result in neuroanatomic aberrations leading to ASD [199]. Evidence has been documented by postmortem studies pointing to the increased apoptotic activity in autistic individuals. These studies found a significantly depleted Bcl-2 content and increased levels of cathepsin D, p53, and caspase-3 in different brain areas of ASD patients [16, 200, 201]. The premature arrest of brain growth in children with ASD has been observed [202], which can also be explained by abnormal activation of apoptosis. Recently, we have performed a systems biology analysis of the SNO-proteome in the *Shank3* InsG3680^(+/+)^ mouse model of ASD [64]. This study revealed a significant SNO enrichment of the apoptotic processes in neurons. We also found S-nitrosylation of the voltage-dependent anion-selective channel protein 2 (VDAC2) in the *Shank3* mutant mice [64]. VDAC2 is an important component of the mitochondrial apoptotic signaling cascade [203] and is activated by SNO [204]. We proposed that SNO-induced activation of VDAC2 may contribute to the mitochondria-related autistic symptoms [64]. Overall, the results of this study are consistent with the above data on the elevated apoptotic activity in ASD that is tightly related to mitochondrial dysfunction [16].

Over the last decade, novel cell death pathways were discovered, including necroptosis, ferroptosis, cuproptosis, *etc*.

#### Necroptosis

In contrast to conventional necrosis, which represents unprogrammed cell death induced by cellular damage, necroptosis is an apoptosis-independent programmed form of necrosis or inflammatory cell death [205, 206]. The signaling pathway of necroptosis has been largely elucidated [207]. This pathway involves the activation of tumor necrosis factor-alpha (TNFα), followed by its receptor TNFR1 stimulation on the cell membrane. TNFR1 binds to several proteins, including tumor necrosis factor receptor type 1–associated death domain (TRADD), TNFR1-associated receptor-interacting protein kinase, also called receptor-interacting protein kinase (RIPK), and TNF receptor-associated factor 2 (TRAF2) [208]. When caspase-8 activity is inhibited, TRAF2 prompts RIPK1 to bind RIPK3 with the formation of a necrosome complex also known as ripoptosome [209]. The ripoptosome phosphorylates mixed-lineage kinase domain-like protein (MLKL), and the phosphorylated MLKL activates mitochondrial ROS production. Then, MLKL translocates to the plasma membrane, causing the loss of membrane integrity and cell death [205].

Bollino et al. [210] have described an alternative calpain-dependent pathway of necroptosis induced by valproic acid (VPA) in the neuronal cell culture. VPA, a histone deacetylase inhibitor, is used for the treatment of mood disorders and epilepsy. However, this drug has been found to exert neurotoxic effects. The study found that this pathway begins with the activation of c-Jun-N-terminal kinase 1 (JNK1) and increased receptor-interacting protein 1 levels (RIP-1). This leads to the cleavage and translocation of AIF from the mitochondrial intermembrane space to the nucleus, phosphorylation of the histone H2A family member H2AX, mitochondrial release of the death-inducing protein Smac/direct IAP-binding protein with low PI (DIABLO), and inhibition of the anti-apoptotic protein X-linked inhibitor of apoptosis (XIAP). This pathway can be inhibited by the cell-survival signaling pathways mitogen-activated protein kinase kinase (MEK)/extracellular signal-regulated kinase (ERK) and phosphoinositide 3-kinase (PI3K)/Akt (protein kinase B), which may protect the cells against the cytotoxic effects of VPA [210]. It is worth mentioning that VPA is also used for generating an environmental model of ASD by injecting this agent into pregnant mice [211, 212].

Necroptosis can also be materialized as a result of activation of the signaling pathways, due to DNA damage, which leads to the release of apoptosis-related factors from the mitochondrial intermembrane space, followed by the activation of the DNA damage–related enzyme poly(ADP-ribose) polymerase (PARP) [213]. In addition, Wang et al. investigated the proximal signaling cascade and found that the mitochondrial phosphatase phosphoglycerate mutase family member 5 (PGAM5) is also involved in various pathways of regulated necrosis via interaction with another mitochondria-related protein dynamin-related protein 1 (Drp1) [213, 214]. A new insight into the role of mitochondria in necroptosis was offered by Zhang et al. [215]. They performed experiments on human hepatic L02 cells treated with CdCl2 and found that Drp1 and retinoblastoma (RB) protein levels were increased and translocated to mitochondria in CdCl_2_-treated cells. Necroptosis and the upregulation of Drp1 and RB were alleviated by the *DNM1L* silencing using a siRNA or pharmacological inhibition of Drp1. The authors concluded that RB directly interacts with Drp1 at mitochondria and forms a complex that enhances the formation of necrosome by binding to RIPK3 [215].

Summarizing the data on the mechanisms of necroptosis, it can be noticed that this kind of programmed cell death requires mitochondrial and cytosolic ROS [216, 217]. Necroptosis is characterized by the excessive accumulation of the products of oxidative stress, such as lipid hydroperoxides [218], end products of glycation [219], and increased activity of bioenergetic pathways, including glutaminolysis [220, 221]. The signaling pathways related to necroptosis include different mitochondria-related proteins, such as Drp1, AIF, PGAM5, and others. Thus, mitochondria play a central role in the mechanisms of this form of programmed cell death [222]. In the context of this review, it is important to note that necroptosis is implicated in ASD pathogenesis. This was recently confirmed by Liu et al. [223]. They carried out a machine-learning and single-nucleus RNA sequencing study of autistic children and reported on the differentially expressed necroptosis-related genes in these patients [223].

#### *Ferroptosis* is another recently discovered iron-dependent process of programmed cell death, [224]

It is genetically and biochemically distinct from other forms of regulated cell death [225]. The mechanism of ferroptosis is based on severe iron-dependent lipid peroxidation and ROS [226, 227]. Ferroptosis does not require a specific pro-death signaling pathway but is triggered spontaneously when the protection of cellular membrane phospholipids from peroxidation by the metabolites and enzymes, including cysteine and glutathione peroxidase 4 (GPX4), are weakened [228, 229]. Gao et al. have found that mitochondria play a central role in cysteine deprivation-induced ferroptosis [230]. They hypothesized that cysteine deprivation impairs the ETC activity and Krebs cycle, leading to hyperpolarization of the IMM followed by lipid peroxidation [230].

Interestingly, cysteine deprivation and activation of necroptosis are associated with the augmented activity of glutaminolysis [231], which fuels the mitochondrial Krebs cycle through the conversion of glutamate to α-ketoglutarate [232]. It has been found that the mitochondrial glutaminase 2 (GLS2), and not the cytosolic GLS1, appears to be involved in ferroptosis [232, 233]. It has also been shown that ferroptosis causes marked morphological changes to mitochondria, such as shrinking, cristae disappearance, disruption of OMM, and increased bilayer membrane density [234, 235]. Finally, some strong ferroptosis inhibitors target exclusively mitochondria [236]. All these facts point to the key role of mitochondria in ferroptosis, particularly because this process is related to cysteine deprivation.

This form of programmed cell death has been identified in various pathologies, including cardiovascular diseases, cancers, kidney diseases, and brain disorders [237, 238]. The accumulating data of animal experimentation involving interventions targeting ferroptosis are promising in terms of the perspective of the symptoms reversal and inhibition of the disease progression [238]. Several studies have found a link between ASD and ferroptosis [239]. Thus, Wu et al. [240] have recently shown that selenium can alleviate autistic behaviors in the BTBR mouse model of ASD by inhibiting ferroptosis via activation of the nuclear factor erythroid 2-related factor 2 (Nrf2)/GPX4 signaling pathway. Another study has identified four key ferroptosis-related genes that might be used as biomarkers for early diagnostics of ASD, although the diagnostic criteria for ASD based on these findings still need to be validated. They also found that piperaquine, an anti-malarial drug, has the potential as a drug for ASD due to its ability to interact with ferroptosis-related genes [241].

Taken together, various cell death pathways exist that in physiological conditions take part in the fine processes of development, differentiation, and removal of damaged or unfunctional cells and subcellular structures, or harmful microorganisms. However, in individuals suffering from different neurodevelopmental disorder conditions, including ASD, these pathways can be hyperactivated, causing irreversible damage to the organism. It is important to note that mitochondria are indispensable in all types of cell death, and consequently, in ASD pathogenesis.

### Mitochondria-related mechanisms of regulation of autophagy and apoptosis in ASD

Normally, the brain maintains a balance between the synthesis and degradation of cellular components in [242]. Removal of the dysfunctional proteins, organelles, and cells is necessary for preserving cellular homeostasis, and this is achieved by the processes of autophagy and programmed cell death, including apoptosis. These processes are essential for human brain development because they represent a quality control system that prevents brain contamination with the toxic products of damaged molecules and cellular structures [243]. It should be noted that in physiological conditions, not only are the processes of synthesis and degradation balanced, but different degradation processes are also finely tuned to ensure the safe disposal of their products. However, the accumulated data indicate dysregulation of autophagy and apoptosis in neurodevelopmental disorders. On the one hand, a growing body of evidence points to autophagy deficiency in ASD [242, 244–246]. On the other hand, mitochondria-related mechanisms of apoptosis activation prevail over the antiapoptotic mechanisms in this disorder [16, 158, 199]. This disbalance results in the accumulation of the toxic products of apoptosis in the brain leading to behavioral abnormalities [158, 243]. Furthermore, many components of the apoptosis and autophagy pathways interact with each other and thus affect each other’s activity and expression [247].

In this section, we discuss the mechanisms of autophagy and apoptosis regulation, and the interplay between these two processes in the context of ASD. The findings on the mitochondria-related signaling mechanisms modulating these processes are presented in Fig. 2.

#### Regulation of autophagy

Autophagy is a process of degradation of the cell components, including proteins and organelles. This process comprises encapsulating debris in double-membrane autophagosomes, followed by fusion of autophagosomes with lysosomes [248]. Autophagy deficiency in neurons results in buildup of ubiquitinated proteins, dystrophy of neural terminals, impairments in synaptic transmission, and eventually, neurodegeneration observed in both neurodegenerative and neurodevelopmental disorders [245, 249, 250]. A post-mortem study by Tang et al. has found autophagy deficiency in the temporal cortices of ASD patients [251]. Importantly, this work also revealed impaired synaptic pruning and ASD-like behavioral phenotype in mice lacking neuronal autophagy. It is now commonly appreciated that impairments in autophagy are implicated in ASD pathogenesis [242, 245, 252].

The origin of autophagosomes in the cell has not been well established. However, the accumulating data indicate that mitochondria are involved in the biogenesis of autophagosomes. In mammals, the key autophagy-regulating proteins, Bcl-2 and Beclin1, are localized to the endoplasmic reticulum and mitochondria [253]. Autophagy is initiated by the autophagy-activating protein complex that includes the UNC-51-like kinase (ULK1), autophagy-related protein 13L (ATG13L), family interacting protein of 200 kDa (FIP200), and ATG101 [254]. In conditions of nutrient depletion, ULK1 is activated, which leads to phosphorylation of the components of the Class III PI3K vacuolar protein sorting 34 (VPS34) protein complex, resulting in the formation of autophagosome and promoting the autophagy flux [255].

The major regulator of autophagy is the mechanistic (also known as mammalian) target of rapamycin (mTOR) signaling, which is closely associated with the activity of mitochondria. Activation of mTOR leads to inhibition of autophagy [248], generally observed in ASD patients [242, 245, 251, 256]. Normally, mTOR is activated in the background of high levels of nutrients and energy substrates [257, 258]. In ASD, however, mTOR appears to be overactivated even at low energy and nutrient levels [176, 251, 259, 260]. The mechanisms of this phenomenon remain unclear. However, the accumulated data suggest that the overactivation of this signaling system could stem from inactivation or genetic ablation of the tuberous sclerosis complex (TSC) [261, 262], the master negative regulator of mTOR. TSC2 forms a heterodimeric complex with TSC1 that suppresses the mTOR complex 1 (mTORC1) activity by inhibiting the small GTPase Rheb, an essential mTORC1 activator [263].

Insufficient ATP production by the mitochondria and nutrient depletion activate the AMP-activated protein kinase (AMPK) [253], which initiates autophagy by phosphorylating and activating the components of the autophagy-activating protein complex and inhibiting the activity of mTORC1. The mTORC1 inhibition by AMPK is accomplished by direct phosphorylation of the regulatory-associated protein of mTOR (Raptor) [264] and indirectly via activation of TSC2 [265, 266]. At high nutrient and ATP levels, AMPK activity is inhibited, and mTORC1 phosphorylates ULK1 and ATG13L of the autophagy-activating protein complex and thus suppresses autophagy [267, 268].

Upon cellular stress, characteristic of ASD [158], ROS production and elevated intracellular Ca^2+^ concentration trigger mPTP opening leading to mitochondrial membrane permeabilization (MMP) [181], initiating apoptosis and necrosis [269], and regulating the activity of autophagy [270, 271]. The MMP results in the loss of Δψ_m_ which prevents the degradation of the voltage-sensitive PTEN-induced kinase 1 (Pink1) [272–274]. This leads to Pink1 accumulation on the OMM, which promotes the recruitment of the E3 ligase Parkin to mitochondria [275, 276]. Parkin ubiquitinates various mitochondrial proteins [253, 277]. It has been suggested that this PTM of the mitochondrial proteins enables the recruitment of the autophagy adaptor p62, leading to the autophagosomal degradation of the damaged mitochondria [12, 278, 279]. In experiments on the human epithelial (HeLa) and SH-SY5Y neuroblastoma cells, Geisler et al. have found that during MMP, p62 may induce autophagy by binding to the ubiquitinated VDAC1 protein localized on the OMM [278]. In contrast, Moscat and Diaz-Meco have shown that p62 activates Raptor of mTORC1 leading to the inhibition of autophagy [280]. Indeed, p62 is a versatile multifunctional protein that can cause opposite effects. On the one hand, it works as an autophagy adaptor, which targets ubiquitinated proteins to the autophagosome for consequent degradation. On the other hand, it acts as an interacting hub for various signaling pathways, including the mTORC1 pathway [281], which inhibits autophagy. Furthermore, p62 is also an autophagy substrate, and it is used as an autophagy marker [280].

Scherz-Shouval et al. have revealed that the antioxidants *N*-acetyl-l-cysteine and catalase abrogated the starvation-induced autophagy of the Chinese hamster ovary (CHO) and HeLa cells [270]. They identified the autophagy protein ATG4 as a sensor of H_2_O_2_ during starvation. The authors hypothesized that H_2_O_2_ formation in the mitochondria results in the inactivation of ATG4, promoting ATG8 lipidation, and thus, activation of autophagy. H_2_O_2_ can also regulate autophagy by modulating the activity of mTORC1. A recent study has shown that the effect of H_2_O_2_ on mTORC1 is dose-dependent. Low doses of this kind of ROS activate while high doses inhibit mTORC1 activity [271].

It is worth mentioning that aberrantly increased levels of NO, RNS, and NO-related PTMs, including SNO, can also significantly affect autophagosomal biogenesis. We have produced a large-scale computational biology analysis of the SNO-proteome in the cortex of *Shank3* mutant mice (a popular model of ASD [171, 282, 283]) and found that among the NO-related molecular alterations, the most prominent change was the activation of the mTOR signaling pathway [171, 176]. Meanwhile, previous studies have reported that NO can disrupt autophagy by the SNO of different proteins, including the components of the mTOR signaling pathway [284].

Cecconi et al. have revealed that the activating molecule in beclin1-regulated autophagy (Ambra1) localized to mitochondria may also contribute to autophagosome formation by interacting with Beclin1 [285]. Experiments have shown that female mice lacking Ambra-1 displayed autism-like behavior, supporting the findings of inhibited autophagy in ASD [286]. The Bax-interacting factor 1 (Bif-1, also known as Endophilin B1) is also an autophagy-activating protein associated with mitochondria [287, 288]. Bif-1 cycles between the cytosol and mitochondria, and during stress it accumulates on the OMM [288], activating autophagosomal biogenesis by interaction with Beclin1 [287]. Bif-1 is also known to activate Bax and Bak during cellular stress, which leads to the activation of apoptosis [289].

Another important player in the regulation of autophagy is the activity-dependent neuroprotective protein (ADNP) [290] discovered by Gozes’ group in 1999 [291]. ADNP and its microtubule end-binding protein motif NAP interact with Beclin1 enhancing autophagy. It has been found that *ADNP* mutation is one of the most frequent genetic causes of ASD [292] resulting in the decreased activity of autophagy [290, 293]. NAP has also been shown to have antioxidative [294] and antiapoptotic properties [295].

The role of mTORC2 in the regulation of autophagy is less investigated than mTORC1. Nevertheless, the studies indicate the mTORC2 involvement in regulating this process. mTORC2 phosphorylates and activates Akt at Ser473. Glucose synthase kinase 3β (GSK-3β) prevents Akt activation via inhibitory phosphorylation of the rapamycin-insensitive companion of mTOR (Rictor). Once activated, Akt, one of the main downstream targets of mTORC2 [296], induces inhibitory phosphorylation of the positive regulators of autophagy Beclin-1 [297], forkhead box O3 (FoxO3) [257], and glial fibrillary acidic protein (GFAP) [298]. mTORC2 also phosphorylates SGK-1 at Ser422, leading to inhibitory phosphorylation of FoxO3 and other activators of autophagy, such as VDAC1 and ULK1 [296]. Meanwhile, mTORC1, in nutrient-rich conditions, activates forkhead box K1/2 (FoxK1/2) which counters Foxo3-induced activation of autophagy by restricting the acetylation of histone H4 and expression of critical autophagy genes [299].

#### Regulation of apoptosis

Mitochondria play a crucial role in the regulation of apoptosis, as reviewed above, and mTOR signaling represents an integral part of these regulatory mechanisms. The mitochondria/mTOR signaling system can induce both inhibitory and activating effects on apoptosis. Thus, mTORC1 promotes the nuclear translocation of the cAMP response element-binding protein (CREB) which stimulates the transcription of the anti-apoptotic gene *MCL-1* in the nucleus and the upregulation of the Bcl-2 family protein myeloid cell leukemia 1 (Mcl-1) [300]. Also, mTORC1 activates the ribosomal protein S6 kinase beta-1 (S6K1) and the eukaryotic translation initiation factor 4E-binding protein 1 (4E-BP1), promoting the translation of *MCL-1* mRNA on ribosomes and the synthesis of Mcl-1 [301]. Mcl-1 inhibits apoptosis through the inhibition of Bax. Also, in the experiments on human breast cancer cells, Won and Seo found that the inactivation of PI3K/Akt/mTORC1 signaling pathway promotes the expression of caspases and Bax leading to increased apoptosis [302]. Cellular stress, often observed in the autistic brain [158], activates p53, which inhibits the activity of mTORC1 via AMPK and TSC2 activation [303]. However, p53 can be activated by mTORC1 [304], causing the translocation of Bax to the mitochondria and initiating apoptosis [303]. Also, p62 recruited to the damaged mitochondria ubiquitinates Mcl-1, leading to its degradation and activation of apoptosis [305]. GSK-3 also mediates the ubiquitination and degradation of Mcl-1. This can be inhibited by mTORC2, leading to the suppression of apoptosis and support of autophagy [306]. Additionally, ATG12 can support apoptosis via direct inhibition of Bcl-2 and Mcl-1 [307]. The opposing effects of mTOR signaling on apoptosis may depend on the distinct pro-apoptotic stimuli. However, these mechanisms warrant further investigation.

Taken together, it can be noted that mitochondria play an integral role in both autophagy and apoptosis, the processes implicated in various forms of brain with neurodevelopmental disorder, including ASD. The accumulated data indicate that mitochondria, mTOR, and autophagy may represent an integral regulatory axis during ASD [16, 251, 253, 308]. Meanwhile, in our opinion, programmed cell death, particularly apoptosis, could be added to this axis because the mechanisms of apoptosis and other forms of programmed cell death are inextricably linked to the mitochondria, mTOR, and autophagy. Many mitochondria-related regulatory proteins and protein complexes display dual functions by affecting the activity of both autophagy and apoptosis. For example, proteins such as p53, p62, GSK-3β, Bif1, Bcl2, Mcl-1, and others regulate both autophagy and apoptosis. The proteins with dual functions are marked in Fig. 2 with an asterisk.

### Mitochondrial dynamics and mitophagy in ASD

Mitochondria are not static, they continuously change their morphology and distribution over the nervous system to match the current needs of the cells [309, 310]. The constant change of mitochondrial shape is referred to as mitochondrial dynamics, which includes fusion and fission. These processes are regulated by specific mitochondrial proteins [310]. Mitochondrial fusion is necessary to repair cell damage, form networks, and exchange genetic information [311]. It is triggered by the GTPases mitofusin 1 (Mfn1) and mitofusin 2 (Mfn2), located in the OMM. These proteins are located on the IMM and interact with the optic atrophy protein 1 (Opa1). Opa1 also participates in cristae formation [312]. It has been found that the knockout of Mfn2 leads to neurodegeneration due to oxidative stress in the brain [313].

The physiological role of fission is to create new mitochondria and to remove damaged and unfunctional parts of mitochondria during severe cellular stress [309, 314]. The process of fission is mediated by the GTPase dynamin-related protein 1 (Drp1). This protein is normally located in the cytosol. When recruited, it becomes oligomerized and is transported to mitochondria where it interacts with protein adaptors, such as the mitochondrial fission factor (Mff), the mitochondrial fission protein 1 (Fis1), the mitochondrial dynamics protein 49 (MiD49/MIEF2), and the mitochondrial dynamics protein 51 (MiD51/MIEF1) [315].

Drp1 undergoes several post-translational modifications. In neurons, cyclin-dependent kinase 1 (CDK1) phosphorylates Drp1 at Ser616 activating mitochondrial fission. Phosphorylation of Drp1 at Ser637 by Ca^2+^/calmodulin-dependent protein kinase Iα (CaMKIα) and protein kinase A (PKA) has an opposite, inhibitory effect on mitochondrial fission [316, 317]. SNO of this protein leads to its phosphorylation at Ser616, and thus, activation of fission in hippocampal neurons [318]. Fragmented mitochondria activate mitophagy, the mitochondria-specific form of autophagy aimed at removing damaged mitochondria [319–321]. Pink1 and the cytosolic E3 ubiquitin ligase Parkin are the key regulators of mitophagy. During mitochondrial stress, Pink1 located in the OMM phosphorylates ubiquitin on Ser65. This results in the translocation of Parkin to the mitochondria and initiation of mitochondrial degradation [322, 323], which helps to maintain neuronal homeostasis [324]. The processes of mitochondrial fusion, fission, and mitophagy are balanced in the normal brain. However, a different picture can be seen in ASD patients.

In a postmortem study of the BA21 temporal cortex of ASD patients, Tang et al. found increased levels of mitochondrial fission proteins, Fis1 and Drp1, and reduced levels of fusion proteins, Mfn1, Mfn2, and Opa1 [79]. These changes in protein expression bring about the fragmentation of mitochondria and their accumulation around the nucleus, which might deprive dendrites and axons of mitochondria [318]. Increased fission caused by the upregulation of proteins Fis1 and Drp1 normally promotes mitophagy [309]. Surprisingly, Tang et al. reported an increase in the levels of dysfunctional mitochondria and mitochondrial membrane proteins, translocase of the outer mitochondrial membrane 20 (Tom20), translocase of the inner mitochondrial membrane 23 (Tim23), and porin in the ASD temporal cortex without a change in the transcription of genes, such as *PARK2*, responsible for the activation of mitophagy. These results indicate that in ASD patients, a preponderance of mitochondrial fission is coupled to impaired mitophagy [79]. Thus, ASD is characterized by an imbalance of the processes of mitochondrial fusion, fission, and mitophagy that leads to contamination of the brain with dysfunctional mitochondrial fragments, insufficient energy supply to the brain tissues, and as a result, impairments of memory and synaptic function [187] contributing to ASD pathogenesis.

### Mitochondrial and synaptic abnormalities in ASD-related syndromes

The growing body of evidence reveals that the ability of mitochondria to adapt to the changing environment and energy demand is impaired in various ASD-related syndromes [325, 326]. This causes the breakdown of the neuronal and synaptic development and function. In this context, let us discuss the most common ASD-related syndromes.

#### *Fragile X syndrome (FXS)* [327] is the most common mutation-based form of intellectual disability

Its prevalence rate accounts for about 1 in 4000-5000 males and 1 in 6000-8000 females [328, 329]. FXS results from an expanded CGG repeat sequence, which may include over 200 repeats (so-called full mutation) in the 5′ untranslated region of the *FMR1* gene situated at Xq27.3. Most females and males carrying FXS have behavioral deficits, including those of ASD [330]. Genetic deletion of the *Fmr1* gene leads to a depletion of fragile X mental retardation protein (FMRP), resulting in the activation of the metabotropic glutamate receptors mGluR [331, 332]. El Bekay et al. [333] found in the brains of *Fmr1*-knockout (KO) mice elevated levels of ROS, GSH, markers of protein oxidation and lipid peroxidation in whole brains, and increased production of NADPH oxidase in the prefrontal cortex, cerebellum, and hippocampus. These data point to the involvement of the *Fmr1* mutation in oxidative stress. Others found decreased Mfn1, Mfn2, and Opa1 levels combined with increased mitochondrial fission in primary neurons from *Fmr1*-KO mice [334]. A study involving the cortex of juvenile and adult *Fmr1-*KO mice has revealed impaired mitochondrial energy metabolism. Activated OXPHOS complexes were found in the isolated cortical mitochondrial membranes. However, ATP production was significantly reduced in these mice [335]. Another study on mitochondria isolated from the forebrain of *Fmr1*-KO mice confirmed the decreased respiratory function at complexes I and II, and the opening of the mPTP. In this work, the authors were able to counter the increased proton leak and mPTP opening by ubiquinone analogs [336]. Huber et al. discovered an elevation in postsynaptic metabotropic GluR type-I (mGluRI) levels in mouse *Fmr1* KO hippocampal neurons. They hypothesized that FMRP downregulates mGluRI. Consequently, loss of FMRP results in an aberrantly increased mGluRI expression in these neurons, leading to enhanced mGluR-related long-term depression causing cognitive impairments and intellectual disability. However, the increase in mGluRs-dependent signaling in the mouse model of FXS was not confirmed in human-based models, and clinical trials with mGluR inhibitors failed to produce a therapeutic effect in FXS patients [331].

Thus, mitochondrial dysfunction has been identified in FXS and its animal models. Some studies conclude on the causal link between the *Fmr1*-KO and abnormal expression of the synaptic mitochondrial proteins. However, the mechanisms underlying the synaptic and mitochondrial aberrations in FXS have yet to be established.

#### Phelan McDermid syndrome (PMS) and Helsmoortel–Van der Aa syndrome (HVDAS)

PMS results from the loss of one functional copy of the *SHANK3* gene of chromosome 22q13 [337]. Shank3 is a scaffolding protein located in the postsynaptic density complex of excitatory synapses. It binds to neuroligins and actin and regulates actin polymerization, growth cone motility, dendritic spine morphology, and synaptic transmission [283]. Therefore, *SHANK3* mutations lead to various symptoms, including behavioral symptoms of ASD. Indeed, deletions or mutations of the *SHANK3* gene have been found both in patients with PMS, which occur in over 50% of ASD patients [338], and in ASD patients outside the PMS. *SHANK3* mutations are likely to cause mitochondrial dysfunction in PMS because six mitochondrial genes, including NADH dehydrogenase 1 alpha subcomplex subunit 6 *(NDUFA6*), cytochrome c oxidase assembly (*SCO2*), tRNA 5-methylaminomethyl-2-thiouridylate methyltransferase (*TRMU*), thymidine phosphorylase (*TYMP*), carnitine palmitoyltransferase 1B (*CPT1B*), and aconitase 2 (*ACO2*), are adjacent to *SHANK3* in the 22q13.3 region [339]. Frye et al. investigated the activity of OXPHOS complexes in the saliva of 51 PMS patients and found abnormal activity of complexes I and IV [339]. Yeunkum Lee et al. have produced a proteomic analysis of synaptosomal Shank3 complexes isolated from the enhanced green fluorescent protein *Shank3* transgenic mice [340]. The cellular components categories of the gene ontology analysis revealed terms like “mitochondrion”, “myelin sheath”, and “cell junction”, indicating the link between Shank3 and mitochondrial proteins [337]. Thus, the association of Shank3 with mitochondrial proteins and processes, including the mitochondrial ETC activity, has been established, although the mechanisms connecting SHANKs and mitochondria at the excitatory synapses remain unknown.

Shank3 can interact with other synapse-related autism-linked proteins. One of these proteins is ADNP [293]. This protein is essential for brain development and function [341, 342]. The neurogenetic syndrome associated with *ADNP* mutation, HVDAS, accounts for 0.17% of ASD cases and is therefore considered a high-risk gene [292]. This gene’s mutations are believed to induce ASD pathology by affecting the chromatin-remodeling ChAHP [343] and SWItch/Sucrose Non-Fermentable (SWI/SNF) complexes [344, 345] during embryonic development. It has also been found that ADNP is involved in histone methylation [346] and acetylation [347] upon interaction with proteins, such as the histone deacetylase sirtuin 1 (SIRT1), which regulates the transcriptional activity and epigenesis [293]. The cytoplasmic ADNP is also known to interact with cytoskeletal microtubules (MTs) and MT-associated proteins, including Tau and MT end-binding proteins (EB1 and EB3), related to ASD pathology [293]. Interestingly, in individuals with HVDAS, the ADNP-SIRT1-EB1/EB3 protein complex may regulate autophagy, which is dysregulated and negatively affects mitochondrial metabolism in autism [344].

Gozes’ group has found that *ADNP* mutations affect MT dynamics and inhibit its interactions with Tau [348, 349], producing a tauopathy-like phenotype manifested in increased GSK-3β activity, Tau hyper-phosphorylation, and cognitive deficiency [293]. Tauopathy is characteristic of the major neurodegenerative disease, Alzheimer’s disease (AD) [349], where tau protein is aberrantly hyperphosphorylated and forms bundles of filaments [350]. Meanwhile, depositions of Tau are also associated with ASD [351]. Interestingly, ADNP mutations were found in postmortem AD olfactory bulbs and hippocampi [348]. These data are consistent with the results of our recent work where we found overactivation of mTORC1 signaling in the cortex of both ASD (*Shank3* InsG3680^(+/+)^) and AD (*P301S*) mouse models [176]. Meanwhile, evidence indicates that mTOR activation augments tau pathology [352]. These somatic and signaling abnormalities are accompanied by increased oxidative stress associated with mitochondrial dysfunction both in ASD and AD [353]. Mitochondrial dysfunction has been recognized as an early pathogenic event for ASD [354] and AD [355], which significantly impairs brain function, as we discuss above. Collectively, there is a tight interplay between the pathogenic mechanisms of ASD and AD.

Ivashko-Pachima et al. have revealed that ADNP protein contains multiple Src homology 3 (SH3) domains crucial for the ADNP-dependent regulation of microtubules. Mutations causing the loss of these sites may adversely affect MT-associated proteins and the interaction of these proteins with Shank3 [293]. This group of researchers has also found that actin plays an important role in ADNP/Shank3 interaction. Both of these molecules contain actin-binding sites. Mutations of *ADNP* and *SHANK3* impair the binding of ADNP and Shank3 to actin leading to autistic behaviors, anxiety, and depression due to synaptic dysfunctions [283, 293]. Thus, these two ASD-related synaptic proteins directly interact via SH3-binding domains and indirectly through binding to actin, determining the crosstalk between PMS and ADNP syndrome.

#### The *DiGeorge syndrome*, also called velocardiofacial or 22q11.2 deletion syndrome (22q11.2DS), is caused by a hemizygous microdeletion (1.5–3 Mb) on chromosome 22

The prevalence rate of this condition is ~1 in 4000 [356]. 22q11.2DS is characterized by neuropsychiatric abnormalities, such as schizophrenia [357], attention-deficit hyperactive disorder, anxiety, depression, and ASD [358].

Importantly, Gokhale et al. have recently shown in a mouse model of 22q11.2DS that mitochondria were damaged in cortical layer 2/3 [359]. These organelles lacked cristae and had high levels of ROS. Systems biology analysis showed that mitochondrial pathways and compartments were strongly related to this pathology. For instance, the authors found that the SLC25A1-SLC25A4 mitochondrial transporter interactome was linked to 22q11.2 gene defect. Moreover, hemideficiency of the SLC25A1 or SLC25A4 orthologues in *Drosophila* appeared to be associated with abnormal synapse morphology, and deficits in synaptic plasticity and neurotransmission [359]. Thus, 22q11.2 deletion, which is related to ASD, is associated with both mitochondrial and synaptic abnormalities. Similar results showing the link between mitochondrial and synaptic dysfunctions affecting synaptic transmission in animal models with 22q11.2 microdeletion were also obtained by other researchers [360, 361].

#### *Rett syndrome* (RS) is caused by mutations in the gene methyl-CpG-binding protein 2 (MECP2)

RS is characterized by language and communication problems, learning and coordination difficulties, and they display autistic-like behaviors [362, 363]. Patients with this syndrome may also have microcephaly and motor difficulties. Children with RS grow slower than typically developing children [364]. A mouse model with exons 3 and 4 deletions of the *Mecp2* gene (*Mecp2B*) appears to have elongated mitochondria in the hippocampal axons and dendrites [365], and increased oxidative stress seen by increased fluorescence of the redox probe roGFP1 both in the mitochondria and cytosol [366]. Oxidative stress in the brain of *Mecp2* mutant mice was also confirmed by another study [367]. The mitochondrial function, as well as long-term potentiation of the hippocampal neurons and astrocytes of *Mecp2*-KO mice, were restored by the ROS scavenger Trolox [187, 368]. It has been found that mitochondria isolated from the cortex and hippocampus of this RS mouse model produce significantly more ROS than those of WT mice [369]. Thus, the clinical and experimental studies convincingly show the involvement of mitochondrial abnormalities in RS, although the mechanisms underlying the role of mitochondrial dysfunction need further investigation.

RS brain does not display obvious manifestations of structural neuronal or glial damage [370, 371]. Therefore, Boggio et al. hypothesized that the neurological symptoms of RS could be associated with abnormalities related to axons, dendrites, and synapses [372]. This hypothesis is consistent with the postmortem examination of the brain of RS individuals. These studies demonstrated a decreased dendritic spine number in the cortex [373] and hippocampus [374]. Importantly, autoradiographic analyses in the basal ganglia and cortex of RS patients showed reduced density of α-amino-3-hydroxy-5-methyl-4-isoxazolepropionic acid (AMPA), N-methyl-D-aspartate (NMDA), and GABA receptors [375]. These results imply that RS is related to aberrations in both excitatory and inhibitory synaptic transmission [337].

Several RS animal model studies also indicated functional, morphological, and molecular changes in synapses in the brain. For example, the frequency of spontaneous excitatory synaptic transmission (EPSCs) in the primary culture of hippocampal neurons of *Mecp2*-KO mice was significantly decreased [376]. Also, autaptic hippocampal cultures produced from these transgenic mice showed a decrease in both amplitude and frequency of EPSCs, whilst neurons of *Mecp2*^*Tg1*^ mice, with an upregulated *Mecp2* gene, had an opposite effect [377]. Following these data, deletion of endogenous *Mecp2* with a specific small hairpin RNA interference reduced spine density after 96 h of expression and decreased the number of mature-shaped dendritic spines [374]. Results of others indicated that down-regulation of *Mecp2* for 5 days decreased neuronal dendritic complexity [378]. Overall, the results of these studies show that *MECP2* gene expression is necessary for normal synaptic development, the balance between the excitatory and inhibitory neurotransmission, morphology, and density of dendritic spines in the brain. Expression and functioning of this gene support mitochondrial function and redox balance. Consequently, mutations of the *MECP2* gene may result in synaptic and mitochondrial aberrations leading to neuropathology, including ASD.

#### *Angelman syndrome (AS)*, *characterized by microcephaly, seizures, motor dysfunction, and mental retardation, is a result of maternal chromosome deletions in the region 15q11-q13 associated with ubiquitin-protein ligase E3A* (UBE3A) critical region [379]

*UBE3A* encodes E6-associated protein (E6-AP), which acts as a cellular ubiquitin ligase and establishes a covalent linkage between a 76-amino acid ubiquitin molecule and its target protein to form a polyubiquitylated substrate [379]. The AS-related E6-AP was found in the nucleus, neuronal synapse, and presynaptic and postsynaptic compartments of the cultured hippocampal neurons [380]. Transcriptome analysis of the mouse cellular models of *Ube3a* deficiency has revealed differential gene expression associated with mitochondrial pathways [381]. Su et al. have reported a defect of complex III of OXPHOS in the brains of *Ube3a* m-\p+ mice. This mitochondrial dysfunction was accompanied by abnormal morphology of the brain mitochondria, and reduced synaptic density [382]. Mitochondrial ROS, particularly O^•–^ levels, appeared to be elevated in the hippocampal neurons of the *Ube3a* m-\p+ mouse model, and CoQ10 analogs could attenuate behavioral deficits in these mice [383, 384].

#### *Cornelia de Lange syndrome (CdLS)* is a rare congenital genetic disorder

Associated symptoms typically include prenatal and postnatal growth delay, a characteristic shape of the craniofacial area, resulting in a distinctive facial appearance, and malformations of the upper limbs. Many infants and children with CdLS have microbrachycephaly. Children with CdLS have autistic features and mild to severe intellectual disability [385]. In 20–50% of cases, this syndrome is induced by a deletion in the *NIPBL* gene on chromosome 5 (locus 5p13) [386, 387]. This gene is responsible for the synthesis of delangin, a protein involved in human development, particularly in the regulation of cohesin complex [388]. CdLS can also stem from mutations on the *SMC3* gene of chromosome 10 [389] and SMC1L1 gene [390]. Hundreds of genes responsible for synaptic transmission, learning, and behavior are deregulated in CdLS patients [391]. Interestingly, it has been found that this syndrome can also be caused by a mutation in the mitochondrial ribosomal protein MRPS22 with deficiencies of OXPHOS complexes I, III, and IV in fibroblast mitochondria [392].

*Smith-Lemli-Opitz syndrome (SLOS)* is an autosomal disorder associated with cholesterol biosynthesis. It is induced by mutations in the gene encoding 3β-hydroxysterol-Δ^7^-reductase (DHCR7), the enzyme catalyzing cholesterol formation by 7-dehydrocholesterol reduction. SLOS patients display multiple anatomic aberrations and intellectual disability, although the phenotype of this syndrome is very diverse. SLOS patients are characterized by cholesterol deficiency and increased levels of cholesterol precursors and their metabolites [393, 394]. Previously, a direct link between impairments of synapse formation and neurite outgrowth was found in an astrocyte culture with impaired cholesterol biosynthesis [395]. Indeed, cholesterol plays an important role in synapse formation and function [396]. Therefore, it is logical to suggest that synaptic dysfunctions occur in individuals with SLOS. Cholesterol production also affects the function of mitochondria. Chang et al. [397] found a significantly increased accumulation of dysfunctional mitochondria in the fibroblasts isolated from SLOS patients under steady-state conditions compared to control cells.

Taken together, mitochondrial abnormalities are closely associated with synaptic aberrations, including impaired synaptic transmission, in various ASD-related syndromes of genetic origin (Table 2). However, the relationships between mitochondria and synapses, and the role of mitochondria in the pathogenic mechanisms of these syndromes have yet to be established.

**Table 2:** Mitochondrial abnormalities in the genetic syndromes associated with ASD.

ASD- related syndrome	Source	Mitochondrial abnormality	References
Fragile X syndrome (FXS)	Brain of *Fmr1* KO mice (a model of the FXS)	• Elevated levels of ROS in • Increased activity of NADPH oxidase • Reduced levels of the mitochondrial fusion proteins MFN1, MFN2, and OPA1 • Reduced ATP production • Decreased ETC activity at complexes I and II • Opening of the mPTP	[333] [333] [334] [335] [336] [336]
Phelan McDermid syndrome (PMS)	Oral cavity of PMS patients	• Disruption of the mitochondrial ETC activity at complexes I and IV	[339]
Helsmoortel–Van der Aa syndrome (HVDAS)	Skin fibroblasts of HVDAS patients	• Reduced mitochondrial respiration	[344]
DiGeorge syndrome (DGS)	Human fibroblasts and mice with the 22q11.2 microdeletion syndrome (models of DGS) Brain of the *LgDel* 22q11DS mouse model (a model of DGS)	• Elevated levels of ROS and decreased expression of the SLC25A1 and SLC25A4 mitochondrial transporters • Impaired mitochondrial and synaptic morphological integrity and increased ROS production • Mitochondrial Ca^2+^ deregulation in brain of a 22q11DS mouse model	[359] [360] [361]
Rett syndrome (RS)	Brain of *Mecp2* KO mice (a model of RS)	• Elongated mitochondria • Abnormal mitochondrial morphology • Increased level of oxidative stress	[365] [365] [187, 367–369]
Angelman syndrome (AS)	Mouse embryonic fibroblasts from *Ube3a*^*-/-*^ mice Hippocampus of *Ube3a m-\p+* mice (a model of AS)	• Altered gene expression profiles of pathways related to mitochondrial functions • Small, dense mitochondria with altered cristae; reduced activity of complex III of the ETC • Increased levels of mitochondrial ROS	[381] [382] [383, 384]
Cornelia de Lange syndrome (CdLS)	A CdLS patient Skin fibroblasts of the CdLS patient	• A homozygous mutation in *MRPS22* gene encoding a mitochondrial ribosomal small subunit protein. • Reduced activities of complexes I, III, and IV of ETC	[392] [392]
Smith-Lemli-Opitz syndrome (SLOS)	Skin fibroblasts of SLOS patients	• Accumulation of dysfunctional mitochondria	[397]

### Conclusive remarks

Accumulating data reveal that the links between mitochondria and the neurodevelopmental abnormalities leading to ASD are multifaceted. This disorder is associated with mitochondrial abnormalities of respiratory function, Ca^2+^ cycling, ROS/RNS production, mPTP opening, activation of different mechanisms of programmed cell death, disbalance of the processes of mitochondrial fusion, fission, and autophagy, and disturbances in synaptogenesis and synaptic transmission which affect the brain development and cause behavioral deficits. In reality, the role of these organelles in autism is hard to overestimate. The fact that mitochondria are essential for various cellular functions but can be affected by different pathogenic factors may explain the similarity of the behavioral phenotype in ASD cases of different origins. Along with mitochondria, synapses are considered end effectors of many molecular mechanisms related to ASD, and the convergence of different neurodevelopmental pathological mechanisms on synapses can at least partially explain the behavioral similarities in different individuals on the spectrum. Meanwhile, as we discussed in this review, synaptic pathology is closely related to mitochondrial dysfunction in ASD. Therefore, mitochondria-associated synaptic abnormalities are likely to contain robust therapeutic targets for ASD that have yet to be discovered. There is still a vast number of “blank spots” in the mechanisms of mitochondria-related autism. Uncovering these spots may lead to the development of novel effective treatments for ASD. This is an important task in light of the ever-growing prevalence of this disorder while no effective pharmacological treatment has been found so far.
